# Calculation of the Intermetallic Layer Thickness in Cold Metal Transfer Welding of Aluminum to Steel

**DOI:** 10.3390/ma12010035

**Published:** 2018-12-22

**Authors:** Zahra Silvayeh, Bruno Götzinger, Werner Karner, Matthias Hartmann, Christof Sommitsch

**Affiliations:** 1 Institute of Materials Science, Joining and Forming (IMAT), Graz University of Technology (TU Graz), Kopernikusgasse 24/I, 8010 Graz, Austria; christof.sommitsch@tugraz.at; 2 Magna Steyr Fahrzeugtechnik AG & Co KG, Liebenauer Hauptstraße 317, 8041 Graz, Austria; bruno.goetzinger@magna.com (B.G.); werner.karner2@magna.com (W.K.); 3 Austrian Institute of Technology (AIT), Light Metals Technologies Ranshofen GmbH (LKR), P.O. Box 26, 5282 Ranshofen, Austria; matthias.hartmann@ait.ac.at

**Keywords:** aluminum-steel blanks, intermetallic layer, cold metal transfer, welding simulation, dissimilar welding, multimaterial car body

## Abstract

The intermetallic layer, which forms at the bonding interface in dissimilar welding of aluminum alloys to steel, is the most important characteristic feature influencing the mechanical properties of the joint. In this work, horizontal butt-welding of thin sheets of aluminum alloy EN AW-6014 T4 and galvanized mild steel DC04 was investigated. In order to predict the thickness of the intermetallic layer based on the main welding process parameters, a numerical model was created using the software package Visual-Environment. This model was validated with cold metal transfer (CMT) welding experiments. Based on the calculated temperature field inside the joint, the evolution of the intermetallic layer was numerically estimated using the software Matlab. The results of these calculations were confirmed by metallographic investigations using an optical microscope, which revealed spatial thickness variations of the intermetallic layer along the bonding interface.

## 1. Introduction

Dissimilar joining of aluminum alloy sheets to steel sheets is an indispensable key process for producing multimaterial car bodies, offering both high crash safety and low vehicle weight. Fusion welding processes in particular have marked advantages regarding the efficient joining of hybrid parts of complex shapes. However, thermal joining of aluminum- (Al) to iron- (Fe) based materials is known to be associated with the formation of intermetallic (IM) Al*_x_*Fe*_y_* phases at the bonding interface [1,2,3]. The formation of these phases is mandatory for bonding of the dissimilar materials, however, excessive formation results in brittleness and therefore in poor mechanical properties of the joints. Thus, controlling the thermodynamically unavoidable interfacial reaction between iron and aluminum is a critical issue regarding the performance of dissimilar joints.

Laboratory experiments have identified that in most cases two main IM phases form at the interface between solid iron or steel and liquid aluminum or its alloys: Al_5_Fe_2_ as the major η-phase [4,5,6,7,8], together with Al_3_Fe (also referred as Al_13_Fe_4_) as the minor θ-phase [9,10,11,12,13,14,15,16,17,18,19,20,21,22,23,24,25,26,27,28,29,30,31,32,33,34]. Some researchers have found additional Al*_x_*Fe*_y_* phases, e.g., AlFe_3_ or AlFe [7,14,15,16]. Dybkov [12] reported ‘paralinear’ growth of the IM phases, meaning that with increasing time the thickness of the Al_5_Fe_2_ phase tends to grow towards a certain limit, while the thickness of the Al_3_Fe phase grows almost linearly after a non-linear initial period. Bouayad et al. [17] also observed that growth of the Al_5_Fe_2_ phase follows a parabolic relationship, but the growth of the Al_3_Fe phase follows a linear relationship. However, according to Bouché et al. [18], both the Al_5_Fe_2_ phase as well as the Al_3_Fe phase exhibit parabolic growth after an initial non-parabolic transient period. The Al_5_Fe_2_ crystals are assumed to have a much higher growth rate than the Al_3_Fe crystals, since the tongue-like Al_5_Fe_2_ sub-layer is generally observed to be markedly thicker than the serrated Al_3_Fe sub-layer.

At least one, or even both, of these two phases was also found to form in dissimilar cold metal transfer (CMT) welding/brazing of aluminum alloys to steel [35,36,37,38,39,40,41,42,43,44,45,46,47,48]. In comparison to conventional gas metal arc (GMA) welding processes, the CMT process is operated with significantly reduced heat input [47,48], which restricts the growth of the Al_x_Fe_y_ phases and therefore limits the thickness of the IM layer [42,43]. The process temperature is high enough to melt the aluminum base material and the aluminum-based filler, but the steel base material remains solid. Thus, dissimilar joining is achieved by a combination of aluminum welding and steel brazing. The single-sided CMT process in particular offers high potential regarding flexible and efficient butt-welding of aluminum alloy sheets to zinc-coated steel sheets, which is of utmost interest in the automotive industry. However, note that the steel sheet can be used in the as-cut condition, i.e., the cutting edge of the steel sheet is uncoated [49].

The growth of the thickness of the IM layer, $x_{IM}$ (m), can be expressed as a function of time, t (s). Diffusion-controlled layer growth, which is assumed as dominant in low temperature and solid state welding processes (e.g., CMT), is commonly expressed using a power-law function:

(1) $$x_{IM}={\left(kt\right)}^{n}$$

For parabolic growth, n = 0.5. The temperature-dependent growth rate coefficient k (m^2^/s) is expected to follow an Arrhenius relationship, where $k_{0}$ (m^2^/s) is the growth constant, Q (J/mol) is the activation energy, T (K) is the absolute temperature, and R = 8.314 J/molK is the gas constant:

(2) $$k=k_{0}\;\exp\left(-\frac{Q}{RT}\right)$$

Table 1 contains values of Q and $k_{0}$ as reported in the literature for calculation of the time- dependent parabolic growth of the major η-phase or of the IM layer, respectively. Both of these constants are usually determined by fitting experimental data captured at different temperatures. Obviously, considerable variations exist between the reported values, which can be attributed to differences in the materials investigated, the experimental conditions, and in formulating the growth equation. Note that most of the experiments have been conducted at laboratory conditions within comparatively narrow temperature ranges. Therefore, the influence of transient or non-uniform temperature fields—as occur, for instance, in most industrial welding processes—on the formation of the IM layer is not considered. Furthermore, if iron or aluminum of technical pureness are used for experiments, the growth constants do not consider the influence of alloying elements, which are known to influence the growth of the IM layer and which are normally present in industrial processes. In particular, increasing the silicon content of aluminum alloys retards IM layer growth [4,10,27,28,29,30,31,32,33,50,51], but increasing the zinc content promotes IM layer growth [4,34,51]. Increasing the carbon content of steels also retards the growth [52,53]. Note that the constants given in References [54,55,56,57,58,59] were determined in experiments where both iron and aluminum were solid (s).

During recent years, different methods have been applied by researchers to model the IM layer, since this layer represents a critical feature influencing the mechanical properties of aluminum-steel joints. Rong et al. [60] conducted thermophysical simulations to clarify reaction mechanisms and growth kinetics at the interface between solid steel and liquid aluminum, and to predict the average thickness of the IM layer. Das et al. [61] proposed a combined theoretical–experimental method, including a numerical model and a set of measured results, to estimate the thickness of the IM layer as a function of key process parameters in a lap joint configuration. Zhang et al. [62] used the Monte Carlo (MC) method to model the growth of IM compounds, and validated their results with bead-on-plate welding of aluminum alloy onto galvanized mild steel.

This work presents a numerical method, which allows fast estimation and three-dimensional (3D) visualization of the IM layer, because the highly irregular microscopic interface between the IM layer and the weld seam is approximated as a smooth surface. 3D visualization of the layer enables the identification of critical weld seam regions where comparatively thin (possibility of insufficient bonding) or thick (brittleness of the joint) IM layers occur. The presented method, which includes (i) calculation of the temperature at the bonding interface between the steel sheet and the aluminum weld by means of finite element (FE) simulation, (ii) validation of the obtained numerical results with temperature curves measured in CMT welding, (iii) prediction of the thickness of the IM layer based on the calculated temperature field, and finally (iv) validation of the predicted thickness with micrographs of weld cross-sections, has already been presented by the authors of this article [63]. This method was also successfully applied by Borrisutthekul et al. [64] for estimating the effect of different heat flow control measures on the thickness of the IM layer in laser welding, and by Mezrag et al. [65] for the indirect determination of the process efficiency in CMT welding.

## 2. Experimental Methods

**Figure 1:**
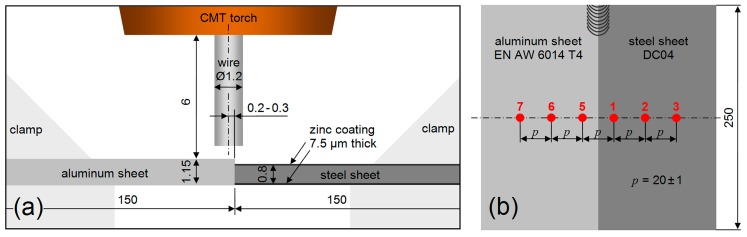
Schematic illustrations of the welding configuration (dimensions in mm): (**a**) front view of the weld butt [46], (**b**) top view showing the positions and the numbering of six thermocouples.

In this study, sheets of aluminum alloy EN AW-6014 T4 were joined with sheets of galvanized mild steel DC04 by means of the single-sided cold metal transfer [47,48] process. The metal sheets were clamped gap-free in horizontal butt-joint position, as schematically illustrated in Figure 1. The dimensions were 250 mm × 150 mm × 0.80 mm for the steel sheet, and 250 mm × 150 mm × 1.15 mm for the aluminum alloy sheet.

The welding equipment included a Fronius CMT Advanced 4000 power source and a Fronius CMT Braze + torch (Pettenbach, Austria), mounted to the arm of a KUKA KR 30-2 robot (Augsburg, Germany). A filler wire of non-commercial aluminum alloy Al-0.3Mg-0.5Sc-0.4Zr was used. Both feeding of the filler wire and supply of the shielding gas were achieved through the welding torch. The main process parameters applied in the welding experiments are summarized in Table 2, and the nominal compositions (wt %) of the materials are listed in Table 3.

## 3. Numerical Methods

The numerical model for calculating the temperature field was created using the ESI Welding Simulation Suite (R 13.5, ESI Group, Paris, France). This software package is based on the Visual- Environment platform, which includes the pre-processing module Visual-Mesh for generating the three-dimensional geometry and the mesh of the model, the module Visual-Weld for defining the welding process, and the post-processing module Visual-Viewer for visualizing the results. The numerical calculation was performed with the finite element (FE) software SYSWELD (R 2017.5, ESI Group, Paris, France).

### 3.1. Geometry and Mesh

**Figure 2:**
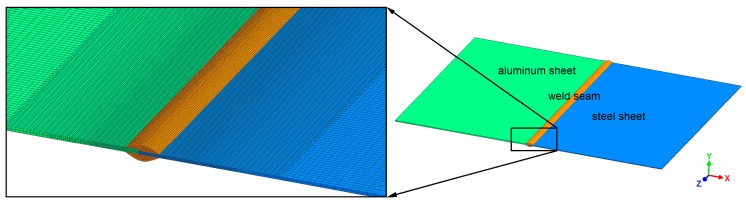
Meshed three-dimensional finite element model.

**Figure 3:**
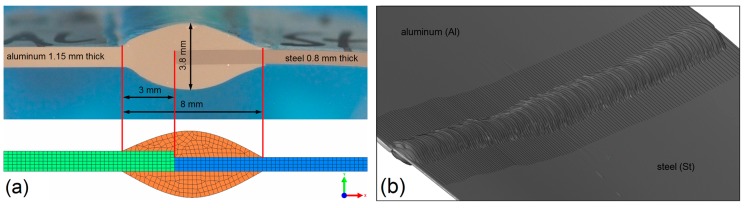
Cross-section of the meshed model based on (**a**) the micrograph of sample 63 and on (**b**) the joint surface reconstruction of sample 62.

As shown in Figure 2, the model included three parts: the aluminum sheet, the steel sheet, and the weld seam, with dimensions according to the experimental welding configuration. The complete model consisted of approximately 504,000 nodes and 425,000 elements. The interface between the weld seam and the steel sheet was meshed with comparatively fine elements, since the temperature at this interface was of primary interest for calculating the thickness of the IM layer. Figure 2 also shows that the mesh was coarser with increasing distance to the weld seam, in order to limit the required calculation time. A cross-section of the meshed model is shown in Figure 3.

According to the results of the welding experiments, the maximum thickness of the weld seam was about 3.8 mm, and the width between the base corners of the weld seam was about 8 mm. These dimensions were estimated from micrographs of the weld cross-section, as exemplarily shown in Figure 3a. The micrographs were captured using a Zeiss Axio Observer.Z1m (Jena, Germany) optical microscope equipped with a Zeiss Axio-Cam MRc5 camera. The dimensions of the weld seam were validated with a three-dimensional reconstruction of the joint surface as shown in Figure 3b, which was captured using the optical 3D scanning system GOM ATOS III Triple Scan (Braunschweig, Germany). Neither small variations of the actual weld seam geometry nor thermal distortion of the sheets were considered in the model, since their effects on the growth and on the thickness of the IM layer are negligible.

### 3.2. Material Properties

**Figure 4:**
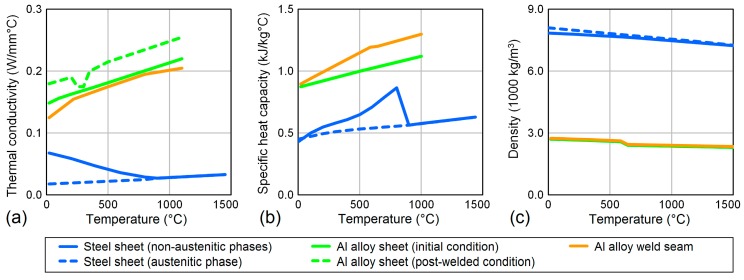
Thermo-physical material properties of the steel sheet, the aluminum alloy sheet and the weld seam: (**a**) thermal conductivity, (**b**) specific heat capacity, and (**c**) density.

As shown in Figure 4, temperature-dependent thermal conductivity, specific heat capacity, and density were considered for both sheets and for the weld seam. These thermo-physical properties were obtained by adapting predefined material data of the available Visual-Weld database (R 13.5, ESI Group, Paris, France).

### 3.3. Process Definition and Boundary Conditions

The main process parameters applied in the welding experiments are summarized in Table 2. Based on the mean welding voltage, U (V), the mean welding current, I (A), and the welding speed, v (m/min), the nominal energy input, E (J/mm), was calculated:

(3) $$E=0.06\frac{UI}{v}$$

The efficiency of the welding process, η, is in the range of about 0.8–0.9 for energy-reduced GMA welding processes with controlled dip transfer [65,66,67]. However, η tends to increase with decreasing arc power [67]. Since the modelled welding process was operated with comparatively low energy input or arc power, η was approximated as unity in the present model. The heat density distribution, q (W/mm^3^), describes the time-dependent movement of the heat source along the pre- defined welding trajectory. It was calculated according to the double-ellipsoidal heat source model of Goldak et al. [68,69]:

(4) $$q\left(x,y,z,t_{w}\right)=\frac{6\sqrt{3}}{\pi \sqrt{\pi }}\;\frac{f\eta Ev}{abc}\;\exp\left(-\frac{3x^{2}}{a^{2}}\right)\;\exp\left(-\frac{3y^{2}}{b^{2}}\right)\;\exp\left(-\frac{3{\left(z-vt_{w}\right)}^{2}}{c^{2}}\right)$$

In Equation (4), x, y and z (mm) are the coordinates of the fixed coordinate system of the model, and $t_{w}$ (s) is the welding time. The linear welding trajectory is oriented in z-direction. The heat source moves with constant welding speed, v (mm/s), along the welding trajectory. The heat source center is located at the initial coordinates $x_{0}$ = $y_{0}$ = $z_{0}$ = 0 when welding starts at $t_{w0}$ = 0. In order to calculate $q_{f}$ and $q_{r}$, the ellipsoidal heat density distributions at the front and rear sections of the weld pool, the factor f is replaced by $f_{f}$ or by $f_{r}$, and the length c is replaced by $c_{f}$ or by $c_{r}$, respectively. The heat fractions $f_{f}$ and $f_{r}$ are deposited at the front and rear sections of the weld pool, with $f_{f}$ + $f_{r}$ = 2. The lengths of the front and rear sections are $c_{f}$ and $c_{r}$. Accordingly, the total length of the weld pool is $c_{f}$ + $c_{r}$, with $c_{f}$:$c_{r}$ = 1:2. The total width of the weld pool is 2a and the penetration depth is b.

The radiative heat transfer coefficient at the surfaces of the weld and of the sheets, $h_{r}$ (W/m^2^K), was calculated based on the Stefan-Boltzmann constant, σ, the thermal emission coefficient, ε, the predefined ambient temperature, $T_{\infty }$ (K), and the local surface temperature, T (K):

(5) $$h_{r}=\sigma \;\varepsilon \;\left(T+T_{\infty }\right)\left(T^{2}+T_{\infty }^{2}\right)$$

In order to quantify the total thermal losses the total heat transfer coefficient, h (W/m^2^K), was then calculated by adding both the radiative heat transfer coefficient, $h_{r}$, and the convective or conductive heat transfer coefficients, $h_{c}$:

(6) $$h=h_{r}+h_{c}$$

The basic input parameters used for the simulation of the welding process are summarized in Table 4. They basically correspond to the conditions of the welding experiments conducted for validating the results of the simulations. However, in order to model the heat losses it was necessary to make some feasible assumptions. Since the temperature dependence of ε was actually unknown, ε = ε(T) was approximated as unity. This is particularly suitable for elevated temperatures, because ε of metals is known to increase markedly with rising temperature, and at elevated temperatures radiative thermal losses become also dominant. The convective and the conductive heat transfer coefficients, $h_{c}$ (W/m^2^K), were assumed to be constant over the entire temperature range. Since the metal sheets were clamped with massive metal bars on both sides of the weld butt, the conductive heat transfer between the sheets and the clamps was certainly higher than the convective heat transfer between the weld seam region and the ambient air.

The dimensions of the weld pool, a, b, $c_{f}$ and $c_{r}$, were calibrated based on micrographs of the joint, as exemplarily shown in Figure 10. Note that in the present work, only the narrow region with the locally molten aluminum sheet was considered for calibration, because the steel sheet remains solid in dissimilar CMT welding/brazing of aluminum alloys to steel. This reasonable simplification allowed the utilization of Goldak’s symmetric heat source model according to Equation (4), even though the aluminum-steel joint had an asymmetric shape.

### 3.4. Calculation of the IM Layer Thickness

The thickness of the IM layer was calculated using the software Matlab (R 2013, MathWorks, Natick, MA, USA). During the non-isothermal welding process, the temperature inside the weld seam and inside the heat affected zone varied strongly with time, T = T(t). Accordingly, the growth rate coefficient varied with time too, k = k(t). Thus, the simple power law function of Equation (1), which is valid for isothermal conditions, cannot describe the growth of the IM layer over the entire welding cycle. However, since assuming T and therefore k as constant is feasible within small time increments, dt, the corresponding growth increment, $dx_{IM}$, can be calculated based on the first derivation of Equation (1):

(7) $$dx_{IM}=n\;k^{n}t^{n-1}dt$$

For discrete steps, and if parabolic growth (n = 0.5) of the IM layer is assumed, Equation (7) can be transformed into Equation (8). For each of these steps, i = 1, 2, 3...m, one can calculate the growth increment, $\Delta x_{IM,i}$, based on the actual time of growth, $t_{i}$, the time increment, $\Delta t_{i}$, and the growth rate coefficient, $k_{i}$, as follows:

(8) $$\Delta x_{IM,i}=\sqrt{\frac{k_{i}}{4t_{i}}}\;\Delta t_{i}$$

**Figure 5:**
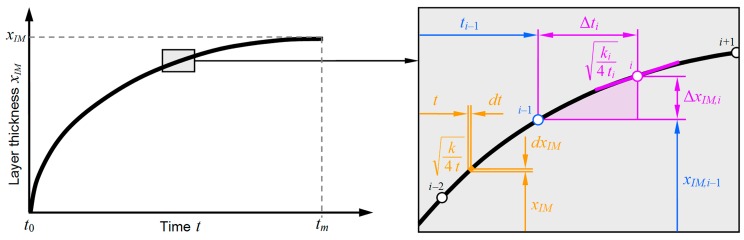
Schematic illustration of the relationship between IM layer thickness and growth time.

Figure 5 illustrates schematically the relationships between the thickness of the IM layer, $x_{IM},$ and the time of growth, t, as well as between the growth increment, $\Delta x_{IM,i}$, and the time increment, $\Delta t_{i}$, as expressed by Equation (8).

Using Equation (9), $k_{i}$ was calculated with the constants Q = 190 kJ/mol and $k_{0}$ = 1.5 m^2^/s, which lay within the range of the values given in Table 1 for the combination of liquid technically pure (i.e., particularly silicon-free) aluminum and solid low-carbon steel:

(9) $$k_{i}=k_{0}\;\exp\left(-\frac{Q}{RT_{i}}\right)$$

Keep in mind that $k_{0}$ is the general growth constant, which is not related to $t_{0}$ = 0. By contrast, the growth rate coefficient $k_{i}$ is certainly related to the time $t_{i}$. The temperature $T_{i}$ was obtained from the finite element simulation, utilizing the constant time increment $\Delta t_{i}$. Note that $t_{0}$ ≠ $t_{w0}$, because $t_{w0}$ = 0 when the welding process starts, but $t_{0}$ = 0 when the local temperature exceeds the limit of $T_{0}$ = 400 °C for the growth of the IM layer. At temperatures below this limit diffusion is quite slow and therefore reactions between aluminum and steel are more or less negligible [5]. According to Equation (10) the total thickness of the IM layer, $x_{IM}$, can be finally calculated by adding all growth increments, $\Delta x_{IM,i}$:

(10) $$x_{IM}=\overset{m}{\underset{i=1}{\sum }}\Delta x_{IM,i}$$

## 4. Results and Discussion

### 4.1. Temperature Field

**Figure 6:**
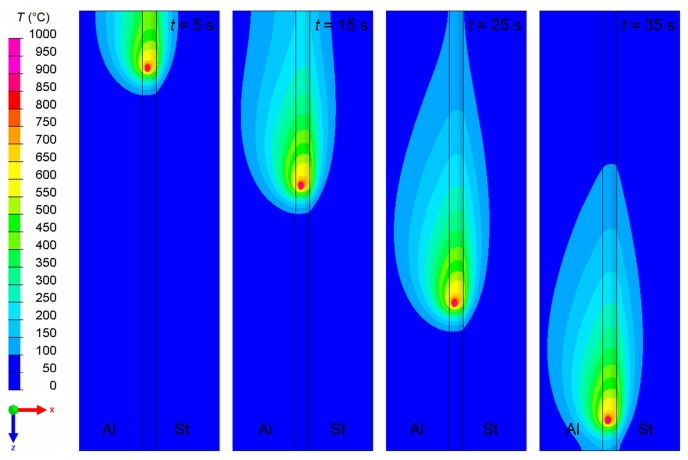
Temperature field at the top surfaces of the aluminum sheet (Al), the steel sheet (St) and the weld seam at 5 s, 15 s, 25 s, and 35 s after starting the welding process.

**Figure 7:**
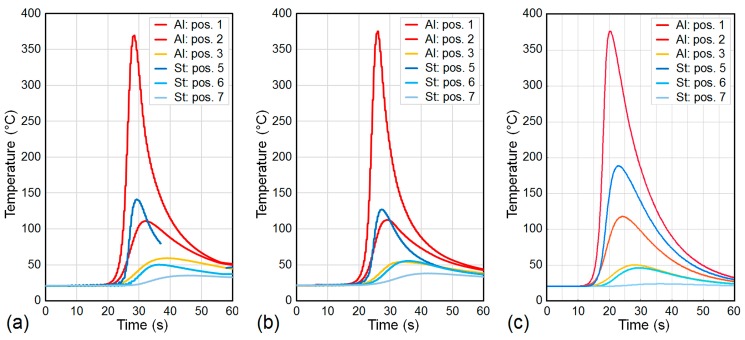
Comparison between temperatures measured using thermocouples during CMT welding of (**a**) sample 62 and (**b**) sample 63, and (**c**) temperatures calculated in the finite element simulation.

The numerical simulation provided the time-dependent temperature field inside the metal sheets and inside the weld seam during the welding process, as well as during the post-welding cooling period. In particular, the evolution of the temperature field next to the weld butt at the top surfaces of the weld seam and of the two metal sheets is illustrated in Figure 6. Though the cooling conditions were equal for both sheets, the non-symmetric temperature field reveals that the majority of the welding heat flowed into the aluminum sheet. This is confirmed by the temperature curves illustrated in Figure 7, which show that the peak temperatures captured at equal distances from the weld butt were almost twice as high in the aluminum sheet as in the steel sheet. Obviously, comparing the temperature curves at particular positions reveals good qualitative and quantitative agreements between the welding experiments (Figure 7a,b) and the welding simulation, (Figure 7c).

The experimentally validated simulation model was used to calculate the temperature not only at the surface, but also at the interface between the weld seam and the steel sheet, which was not accessible for direct temperature measurements. Figure 10a shows that the weld seam contacted the steel sheet at three sections: (i) at the top section, (ii) at the side section, and (iii) at the bottom section. The idealized dimensions of both the top and the bottom sections were 250 mm × 5 mm (i.e., length of the weld seam × mean width of the weld seam contacting the steel sheet), but the dimensions of the side section were 250 mm × 0.8 mm (i.e., length of the weld seam × thickness of the steel sheet). Detailed information regarding the time-dependent temperature field occurring at the three sections of the bonding interface was required to calculate the time-dependent thickness of the IM layer formed during the welding process.

### 4.2. Thickness of the Intermetallic Layer

**Figure 8:**
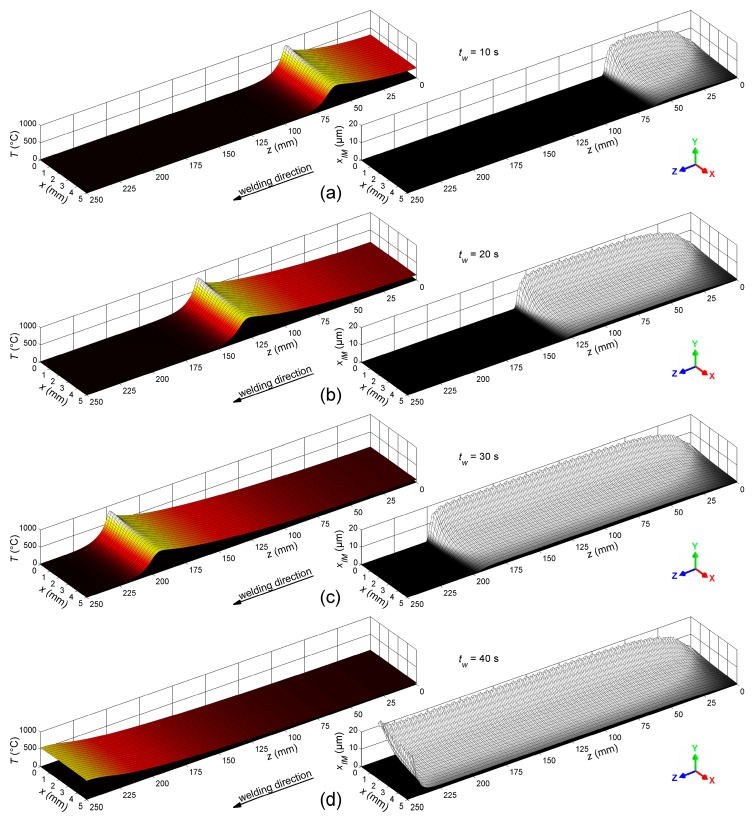
Temperature and thickness of the IM layer, calculated at the top section of the bonding interface between the steel sheet and the weld seam at (**a**) 10 s, (**b**) 20 s, (**c**) 30 s, and (**d**) 40 s after starting the welding process.

Figure 8 shows the calculated temperature, T, (left column) and the corresponding thickness of the IM layer, $x_{IM}$, (right column) at the top section of the bonding interface between the steel sheet and the weld seam at the welding times $t_{w}$ = 10 s, 20 s, 30 s, and 40 s.

**Figure 9:**
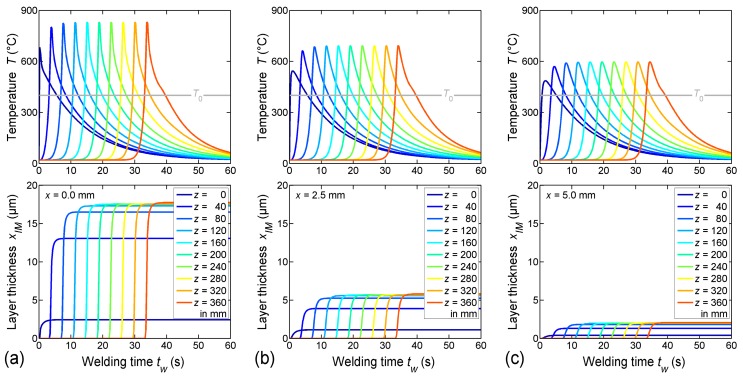
Time-dependent evolution of temperature and IM layer thickness at the top section of the bonding interface (**a**) directly at the weld butt, (**b**) at the center of the interface, and (**c**) at the base corner of the weld seam.

As illustrated in Figure 8, the temperature wave related to the movement of the welding torch propagated in welding direction. The peak of this wave was located close to the actual position of the torch. The growth of the IM layer was initiated when the temperature at the wave front exceeded 400 °C, and it proceeded as long as the temperature stayed above this limit. The thickness of the IM layer in welding direction was constant from z ≈ 50 mm to z ≈ 230 mm, but it varied distinctly perpendicular to the welding direction. Obviously, the thickness did not decrease when the weld cooled down. However, note that the thickness of the IM layer was overestimated at the end of the weld line, since the thermal weld penetration observed in the experiment (i.e., excessive melting of the sheets due to overheating at the end of the weld line) was not considered in the numerical model. Figure 9 illustrates the time-dependent evolution of both temperature and IM layer thickness at ten different positions (z-coordinates) along the welding direction, and at three different distances (x-coordinates) from the weld butt at the top section of the bonding interface.

The grey horizontal line shown in the diagrams of the upper row marks the temperature $T_{0}$ at which the growth of the IM layer was assumed to start in the present numerical model. The diagrams show (a) peak temperatures of approximately 800 °C directly at the weld butt, and (b) peak temperatures of approximately 600 °C at the base corner of the weld seam. Nevertheless, at all positions the temperature decreased about 90% within 60 s. However, the peak temperature strongly influenced the thickness of the IM layer, as shown in the diagrams of the lower row. The calculated thickness was about 17 µm directly at the weld butt, but it was less than 2 µm at the base corner of the weld seam.

### 4.3. Experimental Validation

**Figure 10:**
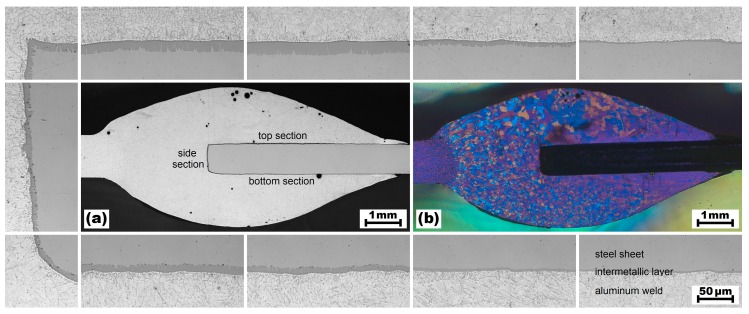
Weld seam cross-section of sample 63, (**a**) unetched and (**b**) etched with Barker’s reagent. The surrounding micrographs illustrate the thickness variations of the IM layer.

In order to validate the results of the calculations, a sample was extracted from the center of the welded blanks at z ≈ 125 mm. This sample was embedded, ground, polished, and prepared for metallographic investigations by means of an optical microscope. The merged micrograph of the entire weld cross-section (both unetched and etched using Barker’s reagent) is shown in Figure 10. As marked in Figure 10a, three sections of the bonding interface between the weld seam and the steel sheet can be distinguished: (i) the top section, (ii) the side section, and (iii) the bottom section. The surrounding micrographs illustrate the varying thickness of the IM layer (dark grey seam between the light grey aluminum weld and the medium grey steel sheet) at different positions on the three sections of the bonding interface.

According to the micrographs shown in Figure 10, the mean thickness of the intermetallic layer was 10.9 µm (SD = 4.2 µm) at the top section, 4.8 µm (SD = 2.1 µm) at the side section, and 7.8 µm (SD = 2.2 µm) at the bottom section. The maximum thickness of approximately 20 µm was found close to the weld butt, at the position where the peak temperatures occurred and where the majority of the filler was deposited. From this point, the thickness of the IM layer decreased bidirectionally: just slightly toward the cutting edge of the steel sheet, but markedly toward the base corner of the weld seam. This was also predicted by the numerical model, as visualized in Figure 8 and Figure 9. However, lack of fusion—which was not included in the present numerical model—occurred at the base corners of the weld seam. The etched micrograph shown in Figure 10b allowed identification of the width of the fusion zone (i.e., the width of the weld pool) between the aluminum sheet and the steel sheet. This width was about 2 mm.

## 5. Conclusions

This work presents a numerical method for predicting the thickness of the intermetallic (IM) layer formed at the bonding interface in dissimilar welding of aluminum alloy EN AW-6014 T4 sheets to galvanized steel DC04 sheets. The method allows fast estimation of the layer thickness and visualization of the layer growth, because the highly irregular microscopic interface between the IM layer and the weld seam is approximated as a smooth surface. Based on the numerical results, which were also validated with single-sided cold metal transfer (CMT) welding experiments, the following conclusions can be drawn:

(1) Due to the steep temperature gradient perpendicular to the welding direction, the thickness of the IM layer varies distinctly. A thickness of about 17 µm was calculated directly at the weld butt, where the peak temperature was about 800 °C, but a thickness of less than 2 µm was calculated at the base corner of the weld seam, where the peak temperature was about 600 °C. These results corresponded well with experimental microstructure investigations.
(2) Excessive formation of the layer was observed at the end zone of the weld seam, where a thermal hot spot occurred. Hence, this region of the weld seam was identified to be critical regarding brittleness of the joint. In comparison, suppressed formation of the layer was observed at the start zone of the weld seam, where the molten filler alloy is deposited onto the initially cold metal sheets. Hence, this weld seam region was identified to be critical regarding insufficient bonding.
(3) In order to obtain a more constant layer thickness, increasing the heat input at the start position of the welding trajectory and decreasing the heat input at the end position of the welding trajectory is advisable. For this reason, automated in-line control of main process parameters, in particular of welding current and welding voltage, is required.
(4) The presented numerical method is based on the temperature field occurring directly at the aluminum-steel interface where the IM layer forms. Thus, the exact experimental or numerical determination of this temperature field is crucial for the subsequent calculation of the layer thickness.
(5) This method represents an engineering approach which is of practical interest for designing dissimilar aluminum-steel weldments, because it enables estimation and/or validation of the basic growth parameters (i.e., Q and $k_{0}$) required for calculating the thickness of the IM layer even for weldments with complex shapes.

## Figures and Tables

**Table 1:** Activation energies and growth constants, as reported in the literature.

Ref.	Researcher	*T* (°C)	Material Combination	*Q* (kJ/mol)	*k*_0_ (m^2^/s)
[5]	Heumann and Dittrich	700–960	pure Fe (s) pure Al (l)	55	-
[8]	Denner and Jones	673–826	0.05 wt % C steel (s) pure Al (l)	170	-
			0.17 wt % C steel (s) pure Al (l)	195	-
[11]	Eggeler, Auer and Kaesche	670–800	low C steel (s) pure Al (l)	134 * 155 **	-
			low C steel (s) Fe-saturated Al (l)	87 * 104 **	-
[17]	Bouayad, Gerometta, Belkebir and Ambari	700–900	pure Fe (s) pure Al (l)	73 * 74 **	-
[30]	Springer, Kostka, Payton, Raabe, Kaysser-Pyzalla and Eggeler	600–675	0.08 wt % C steel (s) pure Al (s,l)	190	-
			0.08 wt % C steel (s) Al + 5 wt % Si (s,l)	17	-
[34]	Springer, Szczepaniak and Raabe, includes data from [4,30,54,57]	400–750	0.08 wt % C steel (s) pure Al (s,l)	190	-
			0.08 wt % C steel (s) Al + 2.5 wt % Zn (s,l)	165	-
[32]	Lemmens, Springer, De Graeve, De Strycker, Raabe and Verbeken	670–725	0.01 wt % C steel (s) pure Al (l)	224	-
			0.01 wt % C steel (s) Al + 1 wt % Si (l)	142	-
			0.01 wt % C steel (s) Al + 3 wt % Si (l)	149	-
			0.01 wt % C steel (s) Al + 10 wt % Si (l)	-72	-
[23]	Tanaka and Kajihara	780–820	pure Fe (s) pure Al (l)	248	1.26 × 10^2^
[29]	Yin, Zhao, Liu, Han and Li	700–800	pure Fe (s) pure Al (l)	207	1.10
			pure Fe (s) Al + 1 wt % Si (l)	169	3.68 × 10^−3^
			pure Fe (s) Al + 2 wt % Si (l)	167	1.46 × 10^−3^
			pure Fe (s) Al + 3 wt % Si (l)	172	1.38 × 10^−3^
[54]	Shibata, Morozumi and Koda	605–655	pure Fe (s) pure Al (s)	226	-
[55]	Jindal, Srivastava, Das and Ghosh	500–600	IF steel (s) pure Al (s)	85	3.82 × 10^−8^
[56]	Kajihara	550–640	pure Fe (s) pure Al (s)	281	1.32 × 10^2^
[57]	Naoi and Kajihara				
[58]	Zhe, Dezellus, Gardiola, Braccini and Viala	535	0.03 wt % C steel (s) Al + 7 wt % Si (s)	153	4.37 × 10^−4^
[59]	Xu, Robson, Wang and Prangnell	400–480	0.08 wt % C steel (s) Al + 0.8 wt % Si (s)	116	-
		480–570		248	-
		400–570		160	-

* maximum thickness of the IM layer, ** mean thickness of the IM layer, (s) solid, (l) liquid.

**Table 2:** Parameters of the welding process.

Parameter	Symbol	Value
Welding current (mean value)	I	71 A
Welding voltage (mean value)	U	8.1 V
Welding speed	v	0.4 m/min
Feeding rate of the filler wire	w	3.9 m/min
Diameter of the filler wire	$d_{w}$	1.2 mm
Distance between the torch and the workpiece	d	6 mm
Angle between the torch and the workpiece	δ	90°
Flow rate of the argon shielding gas	${\dot{V}}_{Ar}$	12 l/min

**Table 3:** Nominal compositions (wt %) of the materials used in the experiments [46].

Material	Al	Fe	Mg	Mn	Si	Cu	Zn	Ti	Cr	V	C	P	S	Zr	Sc
Mild steel sheet DC04 (1.0338)	-	bal.	-	max. 0.4	-	-	-	-	-	-	max. 0.08	max. 0.03	max. 0.03	-	-
Aluminum alloy sheet EN AW-6014	bal.	max. 0.35	0.4–0.8	0.05–0.2	0.3–0.6	max. 0.25	max. 0.1	max. 0.1	max. 0.2	0.05–0.2	-	-	-	-	-
Aluminum filler wire Al-0.3Mg-0.5Sc-0.4Zr	bal.	0.02–0.04	0.2–0.3	0.03–0.05	0.03–0.05	-	-	-	-	-	-	-	-	0.3–0.5	0.4–0.6

**Table 4:** Input parameters used for the simulation of the welding process.

Parameter	Symbol	Value
Nominal energy input	E	86.3 J/mm
Efficiency of the welding process	η	1
Width of the weld pool	2a	2.0 mm
Penetration depth of the weld pool	b	1.5 mm
Length of the weld pool	$c_{f}$ + $c_{r}$	3.0 mm
Duration of the welding process	$\Delta t_{w}$	37.5 s
Duration of the post-welding cooling period	$\Delta t_{c}$	22.5 s
Time increment at time step i	$\Delta t_{i}$	0.25 s
Ambient temperature (= initial sheet temperature)	$T_{\infty }$	20 °C
Stefan-Boltzmann constant	σ	5.67 × 10^−8^ W/m^2^K^4^
Thermal emission coefficient	ε	1
Convective heat transfer coefficient at the weld seam	$h_{c}$	10 W/m^2^K
Conductive heat transfer coefficient at the metal sheets	$h_{c}$	200 W/m^2^K
